# AI-based support for optical coherence tomography in age-related macular degeneration

**DOI:** 10.1186/s40942-024-00549-1

**Published:** 2024-04-08

**Authors:** Virginia Mares, Marcio B. Nehemy, Hrvoje Bogunovic, Sophie Frank, Gregor S. Reiter, Ursula Schmidt-Erfurth

**Affiliations:** 1 Laboratory for Ophthalmic Image Analysis, Department of Ophthalmology and Optometry, Medical University of Vienna, Währinger Gürtel 18-20, 1090 Vienna, Austria; 2 Department of Ophthalmology, Federal University of Minas Gerais, Belo Horizonte, Brazil

**Keywords:** Age-related macular degeneration, Anti-VEGF, Artificial intelligence, Choroidal neovascularization, Deep learning, Drusen, Geographic atrophy, Optical coherence tomography

## Abstract

Artificial intelligence (AI) has emerged as a transformative technology across various fields, and its applications in the medical domain, particularly in ophthalmology, has gained significant attention. The vast amount of high-resolution image data, such as optical coherence tomography (OCT) images, has been a driving force behind AI growth in this field. Age-related macular degeneration (AMD) is one of the leading causes for blindness in the world, affecting approximately 196 million people worldwide in 2020. Multimodal imaging has been for a long time the gold standard for diagnosing patients with AMD, however, currently treatment and follow-up in routine disease management are mainly driven by OCT imaging. AI-based algorithms have by their precision, reproducibility and speed, the potential to reliably quantify biomarkers, predict disease progression and assist treatment decisions in clinical routine as well as academic studies. This review paper aims to provide a summary of the current state of AI in AMD, focusing on its applications, challenges, and prospects.

## Introduction

Artificial intelligence (AI) spans a wide range of techniques within computer science, executing tasks that were traditionally performed by humans [1]. Machine learning (ML) is a branch of AI firstly described by Arthur Samuel in 1959 as the combination of computational science and mathematical concepts used to perform specific tasks without being explicitly programmed [2]. Deep learning (DL) is a class of machine learning techniques dedicated towards developing artificial neural networks with multiple levels of abstraction in which task-specific features are not prespecified by human engineers, but they are learned directly from data using a general-purpose learning procedure [3]. The integration of AI into the field of medicine is not a recent phenomenon. The MYCIN system was developed at Stanford University in the 1970s to assist in diagnosing and treating bacterial infections [4]. From there, the amount of automated algorithms multiplied and more advanced AI-based systems were developed including risk prediction scales that are currently used in clinical practice [5–7]. AI has emerged as a transformative technology across various fields, and its applications in ophthalmology have gained significant attention due to the availability of large digital datasets from retinal imaging. The exponential growth of interest from the scientific community can be easily identified when analysing the raising numbers of publications in recent years. Using “artificial intelligence” and “ophthalmology” as keywords in PubMed there are 3187 publications from 1900 to 2023, 2820 (88%) of them were published in the last 5 years.

Due to the large potential of multi-modal imaging utilized for diagnostics and monitoring of vision-threatening conditions in clinical routine, the retina emerged as the most promising field for application of AI. Age-related macular degeneration (AMD) is one of the leading causes of severe visual loss worldwide and is described as a multifactorial interaction of metabolic, functional, genetic, and environmental factors [8, 9]. Late-stage AMD is characterized by vision-impairing lesions in the macula such as geographic atrophy (GA) in non-exudative AMD or macular neovascularization (MNV) in neovascular AMD (nAMD) [10]. Over the past 20 years, there were significant advances in diagnostic tools, ultimately spectral-domain (SD) optical coherence tomography (OCT) has become the gold standard imaging modality in AMD [11, 12]. 10–15% of all AMD cases advance to nAMD and often suffer from fast progressing devastating visual impairment. Thus, the introduction of intravitreal vascular endothelial growth factor (VEGF) inhibition in 2006 for treating nAMD was a landmark event in disease management [11, 12]. However, the half-lives of these biological drugs are short and in the context of chronic treatment over lifetime, may represent an important burden to the patient and to the health care system [13, 14]. Clinical trials such as PULSAR, TENAYA and LUCERNE analyzed treatment efficacy of more extended regimens in order to address this problem [15, 16]. Furthermore, the first intravitreal complement system inhibitors were approved by the US Food and Drug Administration (FDA) in 2023 to treat patients with GA, [17] representing an additional burden on already stressed health care systems.

Fortunately, efficient AI algorithms relying on high resolution imaging have the potential to reduce time effort and improve quality standards in evaluating disease activity in clinical practice. OCT images are characterized by their high-resolution depiction of retinal structures, containing many millions of pixels in each volume, providing the most critical parameters for guiding treatment decisions in AMD [18]. This review paper aims to provide an overview of the current state of AI in OCT in AMD focusing on its applications, challenges, and prospects.

## AI in retinal imaging

Before the advent of AI-based tools, management of retinal pathologies relied mostly on dichotomous parameters, meaning subjective assessment of presence or absence of specific biomarkers. A binary approach often underestimates biomarker dynamics and the intricate nature of all retinal conditions, including AMD. The multi-modal approaches lead to an enormous amount of information for each patient at each visit. However, traditional methods struggle to capture the subtle variations in pathology, such as atrophy progression, fluid volume and fluctuation. Moreover, novel biomarkers of relevance are subclinical in nature such as photoreceptor layer loss consistent with the ellipsoid layer attenuation. Hence, advanced AI detection tools are needed to reliably inform the clinician about the state of the disease in GA. Nevertheless, the ageing population causes a continuous growth in the prevalence of AMD worldwide, demanding novel strategies capable of screening as well as precisely following disease progression in a faster and reliable manner. AI enables a prompt analysis by quantifying various parameters, that are usually challenging to assess comprehensively by humans in a busy clinical practice [19, 20]. 

Early detection is paramount in managing retinal diseases as it allows for timely intervention in case of conversion to sight-threatening stages of the disease. For example, the presence of large confluent drusen, subretinal drusenoid deposits, refractile deposits, large and central pigment epithelial detachment (PED) and vitelliform material on intermediate AMD were described as phenotype precursors for GA development [21, 22]. Multimodal imaging is a praised gold standard to identify retinal biomarkers. Historically, color fundus photography (CFP) played an important role in screening due to its non-invasive and fast acquisition and broader availability of devices, including the recent emergence of portable handheld devices or applications on smartphones. Due to lower complexity of this imaging modality potentially less intricate algorithms are able to automatically analyze the images [23]. AI-based technology applied on CFP enables the detection of biomarkers such as drusen, haemorrhages and pigment abnormalities and may classify the eye following a binary (referable or non-referable) or multi-class scale (no AMD, early, intermediate, or advanced AMD) [24, 25]. A previous study showed sensitivity and specificity of AI-based screening for intermediate and advanced AMD using CFP achieving 93.2% and 88.7%, respectively [26]. However, limitations including real-world applicability and generalizability as well as demonstrating the long-term benefits on functional outcomes still need further prospective studies. So far, CFP has not been able to provide other than descriptive epiphenomena of AMD.

Fundus autofluorescence (FAF) emerged as a non-invasive imaging modality able to detect light emission from fluorophores such as lipofuscin, supposedly present within the outer segment of photoreceptors and the RPE. This property allowed FAF to further distinguish retinal lesions such as pseudo-drusen and atrophic regions [27]. Usually, blue-light FAF (488 nm excitation wavelength) is most commonly used for imaging AMD, however longer wavelengths, such as green or near infrared, have shown advantages in detecting subtle changes. Recently, AI-based algorithms applied on FAF imaging were developed with the potential of automated segment macular lesions in GA [28, 29]. However, FAF devices are not readily available in clinical practices. Furthermore, the perilesional patterns such as diffusely or focal granular, branching or reticular lipofuscin deposits can be subjectively interpreted and do not reliably correlate with GA lesion progression as neurosensory structures such as photoreceptors are not depicted by FAF.

The amount of information increases by millions due to the pixel-wise extraction when using AI on OCT images [30, 31]. OCT is a non-invasive real-time high-resolution imaging tool of the retina, which, combined with robust algorithms, has the potential to diagnose retinal disease and predict advanced stages, treatment response and visual outcomes in AMD [32]. 

De Faw et al. from the Google group presented the use of automated algorithms in the triage of retinal diseases at Moorfields Eye hospital, determining therapeutic referral need of a patient’s condition. One of the algorithms presented referral recommendations reaching or exceeding the performance of eye care professionals for a range of sight-threatening retinal diseases [33]. The use of this technology on screening and referral pathways may open a cost-effective solution and could increase accessibility in areas of imbalance between caregivers and patients.

Since safety is a key issue in the AI field, the performance of human specialists and algorithms are compared to test and validate automated algorithms. For detection of retinal fluid in AMD patients, an AI-based algorithm showed higher accuracy than eye care professionals, [34] reinforcing the power of these automated measurement tools.

Post hoc analyses offer a good opportunity to validate and refine AI algorithms. However, the utilization of large-scale datasets, such as the Intelligent Research in Sight (IRIS) Registry, may be instrumental in prospectively evaluating AI systems in real-world scenarios [35]. The integration of AI with big real-world datasets not only facilitates the validation of AI technologies, but also contributes to the evolution of precision medicine by tailoring interventions based on the complexities observed in diverse patient populations. Nevertheless, in IRIS, image collection has not yet been integrated into AI and the variability of multiple devices and scan patterns used will present an important obstacle in performing a uniform image analysis.

## AI techniques in OCT analysis

**Fig. 1:**
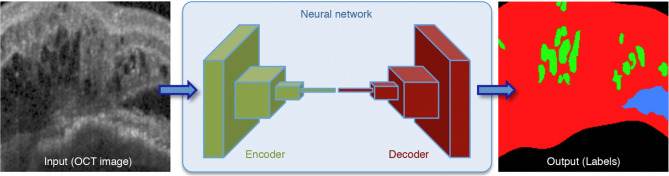
Convolutional neural network with an *encoder*-*decoder* architecture to identify intraretinal fluid (green) and subretinal fluid (blue). The retinal tissue is marked in red. Reproduced with permission from Schlegl et al., 2022 [37]. (For interpretation of the references to color in this figure legend, the reader is referred to the Web version of this article)

**Fig. 2:**
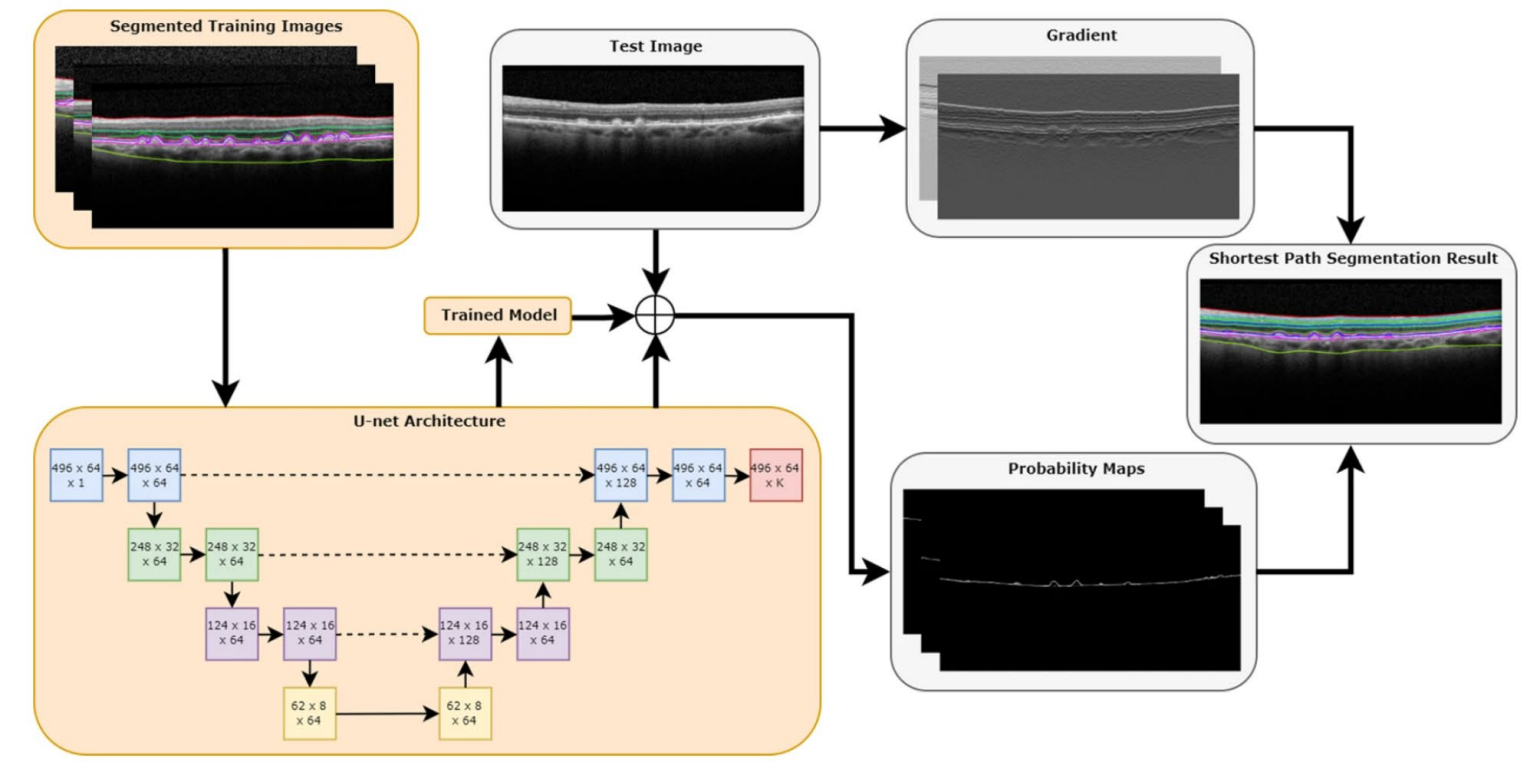
Deep learning utilizing a short-path approach for segmenting retinal layers. Orange boxes represent training steps and gray boxes represent evaluation steps. Reproduced with permission from Mishra et al., 2020 [38]

AI-based OCT analysis typically rely on different DL methods including convolutional neural networks (CNNs) and generative adversarial networks (GANs). CNNs in particular have been at the forefront of AI-driven image analysis in ophthalmology and in retina [36]. They exploit the fact that adjacent pixel values in the image are correlated and they excel at extracting hierarchical features from images, making them particularly effective for the vast amount of high-resolution image data present on OCT. In the context of AMD, CNNs can automatically learn and detect relevant retinal features to perform diverse tasks, such as layer segmentation and fluid quantification (Fig. 1) [37]. For example, Mishra et al. used CNNs to develop a shortest-path algorithm of 11 retinal layers, drusen and subretinal drusenoid deposits in SD-OCT based on probability maps (Fig. 2) [38]. Transfer learning, which involves fine-tuning pre-trained CNN models on OCT data, has proven beneficial for small datasets by leveraging knowledge gained from larger related datasets.

Layer segmentation is a critical step in extracting anatomical structures from OCT images. AI-powered segmentation algorithms employ techniques like U-Net, a convolutional network architecture tailored for segmentation tasks. Indeed, a precise segmentation aids in identifying disease-specific features such as photoreceptor integrity, defined as continued segmentation of the area between the top of the ellipsoid zone and the outer boundary of the interdigitation zone, and RPE layer disruption, enabling more accurate diagnosis and disease progression assessment [39–41]. 

Furthermore, GAN is a deep learning method that consist of two neural networks, a generator, and a discriminator, automatically working in tandem to produce high-quality synthetic data. In OCT analysis for AMD, GANs can aid in data augmentation for addressing the challenge of imbalanced datasets and for training models to detect abnormalities on OCT without the need for manual labelling [42]. GAN-generated images can also be applied for super-resolution and denoising in OCT images [43]. 

The foundation model’s ability to leverage vast datasets for nuanced pattern recognition can play an important role in the early detection of retinal diseases [44]. A self-supervised learning-based foundation model was recently described as capable of training on unlabelled retinal images and showing satisfactory performance in detecting ocular disease and predicting systemic disease based on retinal imaging. Furthermore, it holds promise for democratizing access to medical AI and advancing clinical implementation by providing a publicly available resource for further research and applications [44]. 

### AI in OCT in intermediate AMD

Patients with early and intermediate AMD frequently show few to no symptoms. The speed of progression to advanced stages varies widely, hence it is of great importance to accurately identify key biomarkers and to predict the patient’s conversion, as vision loss occurs only in advanced stages with foveal destruction. Since early interventions with intravitreal therapies are more beneficial in preserving visual function of patients, there is a strong drive towards early detection of converters [45]. Multiple prognostic markers have been identified preceding conversion to advanced AMD including demographics, genotype and structural features. For example, the location, volume and size of drusen indicated the progression to nAMD, whereas outer retinal thinning led to GA [46]. However, the features are confluent suggesting a common mechanism among the different types of AMD.

Subretinal drusenoid deposits (SDD) have been shown to be a significant biomarker, associated with a higher risk for developing type 3 MNV, outer retinal atrophy and GA [47, 48]. Therefore, it is desirable to automatically quantify them and implement in predictive progression models. However, segmentation of SDD can be challenging. A previous study showed an overall substantial inter-reader agreement regarding the presence of SDD, but a slight and moderate inter-reader agreement for presence of type 1 and type 2 SDD on selected OCT B-scans [49]. Furthermore, a larger difference between human graders and AI was reported for SDD when compared to drusen [38]. With targeted efforts in this field, automated SDD detection is advancing.

The ongoing PINNACLE trial consisting of multimodal imaging including over 400.000 OCT images from AMD patients with a follow-up of up to 3 years is conducted to characterize and validate biomarkers for conversion and, as secondary outcome, develop predictive risk models [50]. Recently, a DL classifier was developed using data from the PINNACLE study identifying normal eyes and the onset of early and intermediate AMD, GA and nAMD automatically. It consists of a two-stage CNN categorizing disease stages with an AUC of 0.94 in a real-world test set [51]. Based on multiple risk factors for conversion identified, ML algorithms were developed to predict nAMD or GA conversion in fellow eyes of nAMD patients [52, 53]. Using the HARBOR trial dataset, an algorithm showed that converters to nAMD present different patterns than GA converters such as thickening of the RPE drusen complex, increased drusen area, HRF and outer nuclear layer (ONL) thickening in areas with hyperreflective foci. GA-converters on the other hand show global ONL thinning, RPE and inner segment/outer segment junction (IS/OS) thinning and hyperreflective foci in the ONL. This predictive model achieved an AUC of 0.68 with 0.46 specificity and 0.80 sensitivity for nAMD and a higher AUC of 0.80 with 0.69 specificity and 0.80 sensitivity for GA prediction [53]. 

### AI in OCT in nAMD

Automated tools for guiding anti-VEGF therapy in nAMD is not new. Central subfield thickness (CST) is an automated measurement tool, widely used as endpoint and guide for treatment decisions in many clinical trials [14, 54]. However, this parameter does not provide any detailed information about location and extension of disease-specific activity and is often not adequately aligned in diseased macula. Furthermore, it does not distinguish between neurosensorial layers, retinal fluid compartment and pigment epithelial detachments. Previous studies have shown a weak correlation between CST and visual acuity as well as between CST and retinal fluid volumes [55, 56]. Moreover, in recent clinical studies comparing durability of the novel substances CRT values were used in various and irreproducible combinations making an objective comparison of the novel therapeutics impossible.

The most recent AI-based algorithms developed for OCT involve biomarkers such as fluid volume quantification in each compartment (intra- and subretinal), fibrovascular PED, subretinal hyperreflective material (SRHM) and hyperreflective foci [10, 19]. Despite the importance of all high-order biomarkers on disease classification and progression, [57, 58] post hoc analysis from TREND study and from the Fight Retinal Blindness! dataset using DL and ML showed that retinal fluid is still the most important anatomical biomarker for predicting disease activity, treatment demand and visual outcomes in nAMD [59, 60]. Indeed, recent analysis showed that not only the location of fluid is important for disease progression, however the dynamic fluctuation of each retinal fluid has a high impact on the outomes [61]. Volumes and changes in volumes of retinal fluid have a substantial impact on vision outcome. Higher IRF and PED were associated with worse visual outcomes, despite IRF being the fluid type with faster response to anti-VEGF therapy. SRF showed slower resolution, intuitively leading to an increased number of injections during the first year in a pro-re-nata regimen, however no significant correlation with worse functional outcomes was found presumably due to the predominant location of SRF outside of the central 1 mm of the fovea [59, 60, 62]. This highlights the notion that traditional treatment patterns have to undergo a reality check by a rigorous structure/function correlation. In long-term follow up, retinal fluid volumes and visual acuity are not generally, but individually correlated, indicating a concomitant neurodegenerative process [63]. Furthermore, despite “regular” treatment, most of nAMD patients are prone to develop subretinal fibrosis and macular atrophy with time [64, 65]. Higher amounts of fluid, particularly volume fluctuations, the presence of subretinal hyperreflective material and MNV type revealed to be correlated with atrophy or fibrosis development [61, 66–68]. Photoreceptor loss and RPE loss were strongly correlated with development of macular atrophy [69]. Thus, to better understand late anatomical outcomes, early changes on the photoreceptor layers in association with fluid behaviour may be further investigated through precise in vivo measurments [70]. Furthermore, there is still an open question if SRF, overall or dependent on its dynamics, might be protective against macula atrophy development in patients with nAMD [71]. 

OCT angiography (OCTA) represents a powerful non-invasive technology able to analyse choroidal and retinal vessels, including MNV characteristics in nAMD. A previous study analysing MNV characteristics using OCTA found a correlation between higher vessel tortuosity within the MNV area and worse visual outcomes as well as stronger trend to atrophic changes, despite lower exudation at baseline [72]. In the other hand, Sulzbacher et al. reported no significant correlation between OCTA patterns such as vessels density of the neovascular lesion and BCVA [73]. An automated and precise tool applied to choroidal flow, vessel density and other MNV characteristics may open new perspectives on vascular biomarkers.

### Home monitoring OCT

Home monitoring OCT has emerged as an innovative technological paradigm aimed at optimizing the surveillance of individuals affected by chronic sight-threatening pathologies, notably those requiring recurrent monitoring and fast intervention, such as nAMD [74]. Home screening tests for nAMD are not a novelty. Amsler grid and preferential hyperacuity perimetry has been previously proposed to detect metamorphopsia as an early sign of choroidal neovascularization in AMD patients [74, 75]. The current development of an AI-based fluid monitoring algorithm implemented on a home OCT device (The Notal Vision Home OCT system) showed promising results with feasible self-scan rates. This technology is not yet available on the market, however it showed feasibility in early detection of biomarkers and advanced stages of AMD such as neovascularization [76]. Home monitoring OCT may bring advantages including a decreased number of visits to the eye hospital and close monitoring of disease progression. However, there are challenges such as quality of data acquisition, safety of data transfer and integration of the acquired data into a local healthcare system. A previous economic evaluation based on a simulation showed that a home visual-field monitoring system for early CNV detection was cost-effective compared with scheduled examinations alone on patients at risk of developing nAMD [77]. Further cost-effective simulations should be conducted for new devices. Additionally, the concomitant escalation in data volume stemming from increased imaging monitoring frequencies is a major issue. Also, compliance has always prevented reliable and long-term self-monitoring in chronic disease. Shared-care as already implemented in Great Britain with community-based monitoring “around the corner” by fully equipped opticians may be a more realistic model, particularly if supported by standardized AI-based detection tools.

### AI in geographic atrophy

GA is characterized by degeneration of the photoreceptors and RPE, accompanied by degeneration of the subjacent choriocapillaris, leading to irreversible vision loss [78, 79]. The literature mentions that GA affects around 5 million people worldwide, [80] however this number might be underestimating the total number of patients, as it primarily accounts for fovea-centered lesions. Consequently, it overlooks a significant spectrum of the disease in earlier stages all arising from the perifoveal area and being non-symptomatic. The diagnosis of GA was initially based on fundus photography [81]. Blue-light fundus autofluorescence (FAF) emerged as a valuable tool in diagnosing GA, since the degeneration of the RPE results in a clearly demarcated area of hypo-autofluorescence. Near-infrared reflectance (NIR) imaging, which has a longer wavelength, has also provided a benefit for visualization of GA lesions with lower interference caused by the macular luteal pigments [28, 82]. Therefore, the number, location and size of GA lesions as well as other disease-specific biomarkers could be measured easier with FAF [83]. Therefore, FDA and European Medicines Agencies accepted FAF-based measurements of changes in GA area as anatomical endpoint in the early clinical trials [84, 85]. Availability of high-resolution three-dimensional OCT imaging together with AI tools detecting the pathognomonic neurosensory, yet subclinical features not accessible to human specialists by retinal images alone, but accurately visualized on OCT-based AI analysis represent the novel horizon of precision medicine [28, 29, 86]. 

**Fig. 3:**
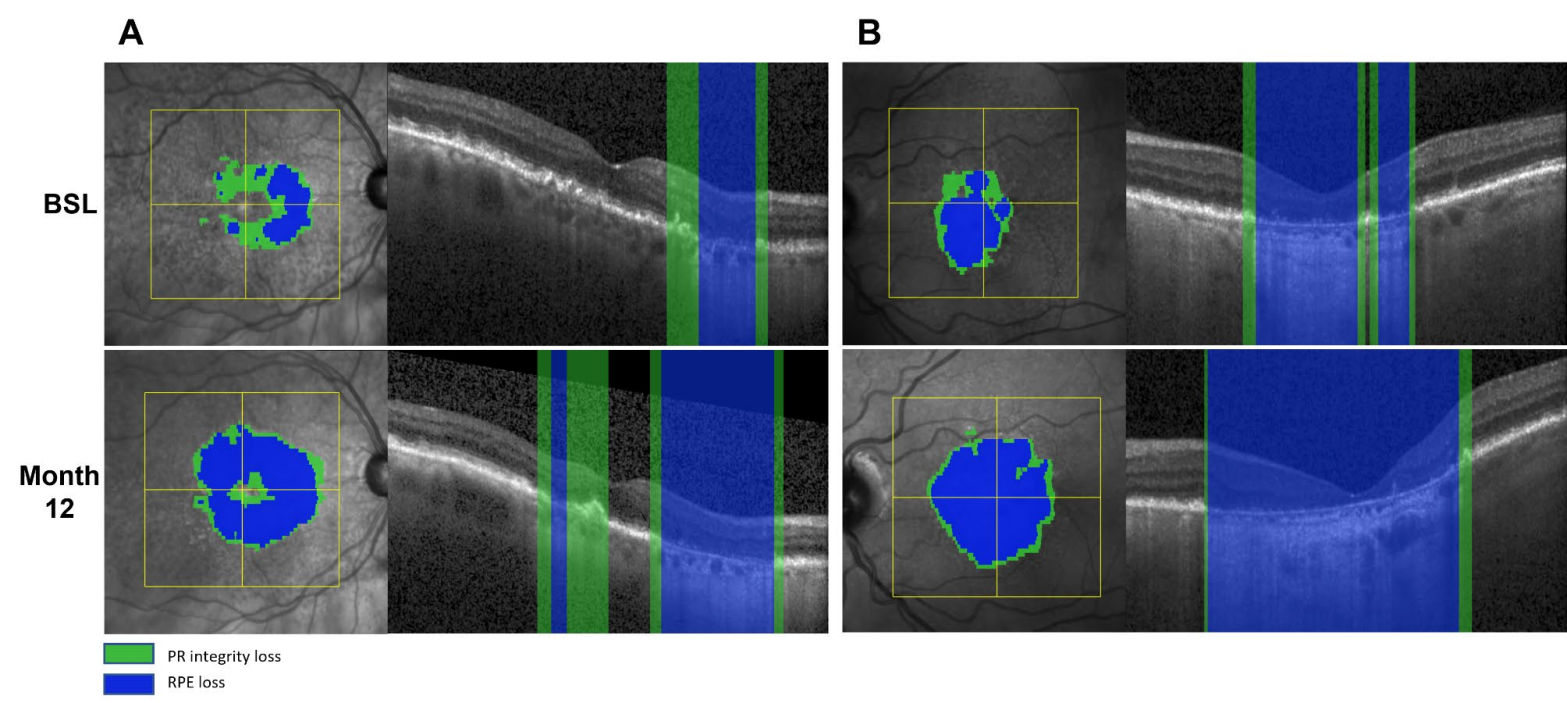
Geographic atrophy (GA) lesion from two patients at baseline (BSL) (upper row) and at month 12 (lower row) from OAKS clinical trial dataset automated segmented with the AI-based GA monitor. Retinal pigment epithelium (RPE) loss (blue) and photoreceptor integrity loss (green) are shown as en face visualizations (left) and example B-scans (right). RPE loss extends into regions of preexisting PR loss

**Fig. 4:**
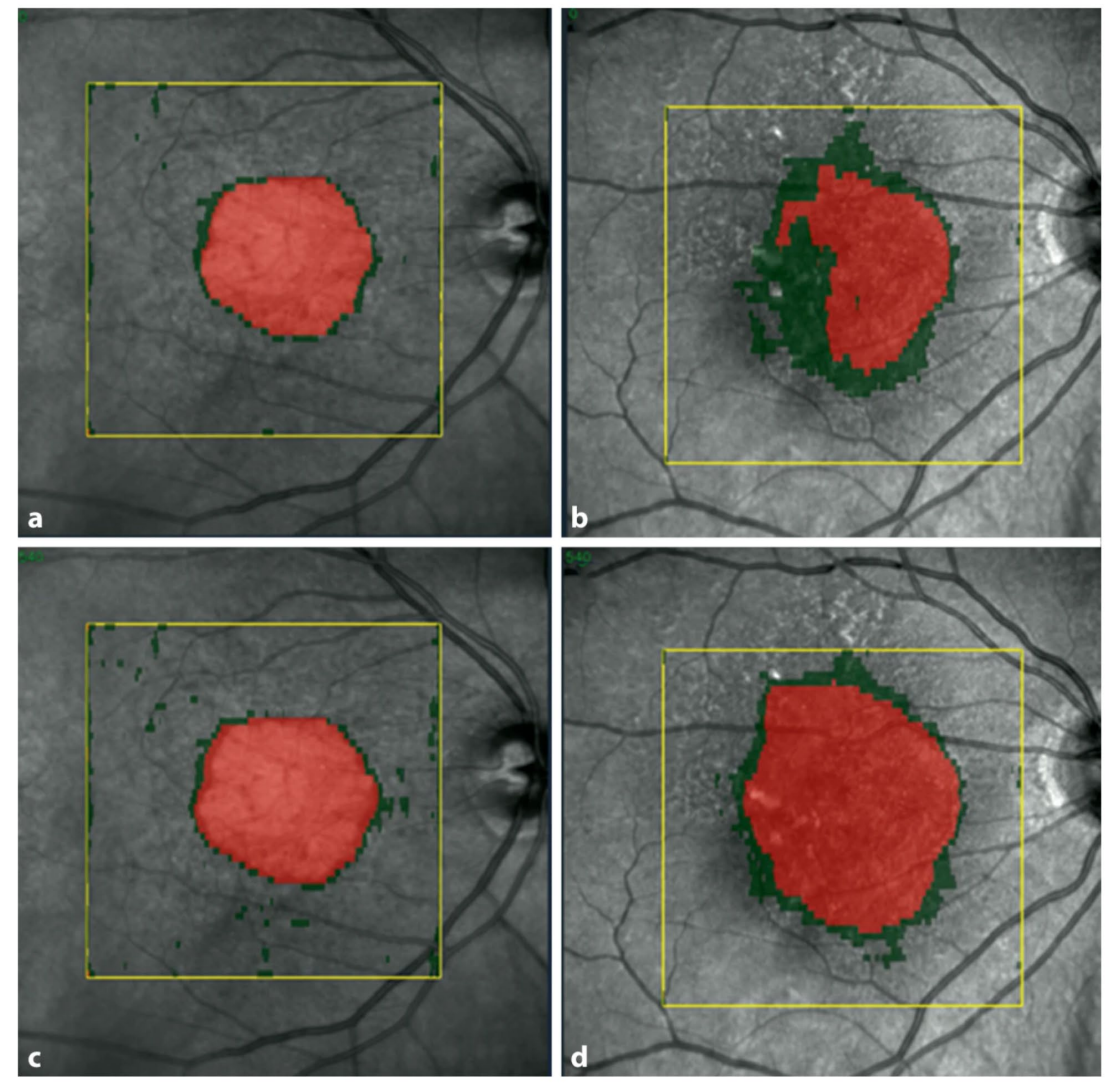
Example of 2 lesions with geographic atrophy and different PR/RPE loss ratios. PR loss is marked in green and RPE loss in red. (**a**) shows a lesion with a small PR/RPE loss ratio and (**b**) a lesion with a large PR/RPE loss ratio at baseline. Letters (**c**) and (**d**) show the respective lesions at month 12 with significantly faster growth in the lesion with the higher ratio (**d**). Reproduced with permission from Schmidt-Erfurth et al., 2023 [90]

In clinical practice, OCT devices are widely available and are the gold standard in monitoring AMD. SD-OCT provides a more detailed information of the condition of the outer retinal layers, including the degeneration of photoreceptors in the lesions’ active junctional zone [47, 87]. Therefore, OCT might provide more insights into progression patterns due to the detailed visualization of photoreceptor alteration. In a post-hoc analysis of the FILLY phase 2 clinical trial data set, findings from OCT imaging were consistent with FAF results measuring the RPE defect. Both imaging modalities could prove the superiority of treated patients regarding RPE loss, but only three-dimensional assessment in OCT was able to reveal early superior maintenance of photoreceptor integrity with complement inhibition [88]. To precisely measure these parameters, a DL algorithm segmenting A-scan regions on SD-OCT was clinically validated and tested using study datasets as well as real world images (Fig. 3) [88]. The GA monitor computes topographic maps and measurements of RPE and photoreceptor integrity loss based on 3D imaging and is accessible by a simple upload of a standard OCT image to the Heidelberg engineering Spectralis AppWay [41]. In the consecutive phase 3 studies, Oaks and Derby, high statistical significance with *p* < 0.0001 was provided demonstrating that disease activity, i.e. GA growth correlated with the ratio between photoreceptor integrity loss and RPE loss, i.e. the PR/RPE loss ratio. The PR/RPE ratio also strongly determined the level of therapeutic benefit in the study results qualifying as a most reliable clinical parameter for treatment indications in GA (Fig. 4) [89, 90]. 

As the question remains unclear which patients should be treated, clinicians search for predictors of disease activity. For this purpose, a validated algorithm was used to predict topographic progression of GA by analysing RPE loss, photoreceptor integrity and hyperreflective foci (HRF) [53]. Higher progression rates of GA were associated with atrophies closer to the fovea, HRF at the junctional zone and thinner photoreceptor layers. These tools could help in identifying progression patterns, which would support clinicians to better identify patients, who would benefit from a potentially life-long treatment.

### AI-based OCT tools in clinical trials

Despite the previously mentioned potential of using AI in clinical practice, another evident application pertains to the recruitment processes in clinical trials for AMD. Developing a new drug presents a financial challenge, attributable to regulatory requirements, extensive data collection and usually prolonged timelines associated with clinical trials. The use of ML and DL models exhibit substantial promise in accelerating the enrolment of participants who are more likely to present faster progression to advanced stages of the disease, may present stronger response to specific novel therapies, and demonstrate a reduced likelihood of premature withdrawal from a trial [91]. An efficient selection process can increase sample size and consequently the detection of statistically significant differences between groups on a trial [53]. On the other hand, selecting patients with higher disease activity and possibly stronger response to a specific therapy could improve patient enrolment in clinical trials. Additionally, automated algorithms can assess morphological endpoints in a precise and reliable manner, most importantly in real-time for all clinical sites providing highest image quality as the images immediately undergo analysis control. Especially in GA, functional endpoints such as best-corrected visual acuity are frequently insufficient to describe all aspects of visual impairment [83]. Structural biomarkers are less influenced by patient compliance than functional endpoints. Clinical trials have been performed to study how disease activity assessment is influenced and supported by AI-based enrichment of OCT images. RAZORBILL (NCT04662944), Notal Vision Home OCT study (NCT04642183), and a prospective study using fluid monitoring (NCT05093374) are examples of ongoing trials using automated segmentation of retinal fluid volumes. A clinical trial using a DL algorithm on OCTA (NCT05969418) to analyze neovascular membrane vessel characteristics has started and as previously mentioned in this review the PINNACLE clinical trial cohort is being conducted for AI-based segmentation of early atrophy and fluid-related biomarkers on OCT (NCT04269304). Less intricate algorithms based on CFP segmentation are also under investigation for screening purposes such as iPredict (NCT04863391) and VeriSee AMD (NCT05593913).

## The limitations of AI-based algorithms

The implementation of AI-based algorithms in AMD diagnosis and treatment comes with the known limitations of innovative approaches. The demand of large datasets for training AI models may introduce biases, as certain demographics or variations in disease characteristics may be underrepresented. The ethical considerations surrounding patient privacy, data security, and the interpretability of AI decisions also raise concerns and must comply with different regulations in each nation. Another aspect to consider are adequate reimbursement models. Furthermore, the diversity of devices, launch of new imaging machines, and variations in imaging protocols across different clinical settings can hinder the development of universally applicable algorithms. While AI in retinal images holds promise, addressing these limitations is crucial to realizing its full potential in enhancing diagnostic accuracy and ensure its effective and ethical integration into clinical practice. The overwhelming introduction of OCT imaging hand-in-hand with anti-VEGF therapy using OCT hardware as a “fluid meter” clearly serves as a potent role model for the introduction of OCT-based AI analysis in times of GA management, an even bigger responsibility, 20 years later.

## Conclusion

AI-based models can strongly benefit clinical practice and research in AMD. Multimodal imaging, the conventional standard for diagnosing patients with AMD, is being replaced by OCT imaging. Furthermore, DL and ML algorithms showed the potential of reliably quantifying biomarkers, predicting disease progression and assisting treatment decisions. The next steps have to be taken for bringing AI from clinical research to its clearly needed application in everyday clinical practice, however, ongoing advances in the field are steadily narrowing this gap.
